# The arrhythmic substrate of hypertrophic cardiomyopathy using ECG imaging

**DOI:** 10.3389/fphys.2024.1428709

**Published:** 2024-08-14

**Authors:** Ji-Jian Chow, Kevin M. W. Leong, Matthew Shun-Shin, Sian Jones, Oliver P. Guttmann, Saidi A. Mohiddin, Pier Lambiase, Perry M. Elliott, Julian O. M. Ormerod, Michael Koa-Wing, David Lefroy, Phang Boon Lim, Nicholas W. F. Linton, Fu Siong Ng, Norman A. Qureshi, Zachary I. Whinnett, Nicholas S. Peters, Darrel P. Francis, Amanda M. Varnava, Prapa Kanagaratnam

**Affiliations:** ^1^ National Heart and Lung Institute, Imperial College, London, United Kingdom; ^2^ Cardiology Department, Imperial College Healthcare NHS Trust, London, United Kingdom; ^3^ Cardiology Department, Barts Heart Centre, London, United Kingdom; ^4^ Cardiology Department, Queen Mary, University of London, London, United Kingdom; ^5^ Cardiology Department, Oxford University Hospitals NHS Trust, Oxford, United Kingdom

**Keywords:** hypertrophic cardiomyopathy, sudden death, electrocardiographic imaging, implantable defibrillator, risk stratification

## Abstract

**Introduction:** Patients with hypertrophic cardiomyopathy (HCM) are at risk for lethal ventricular arrhythmia, but the electrophysiological substrate behind this is not well-understood. We used non-invasive electrocardiographic imaging to characterize patients with HCM, including cardiac arrest survivors.

**Methods:** HCM patients surviving ventricular fibrillation or hemodynamically unstable ventricular tachycardia (n = 17) were compared to HCM patients without a personal history of potentially lethal arrhythmia (n = 20) and a pooled control group with structurally normal hearts. Subjects underwent exercise testing by non-invasive electrocardiographic imaging to estimate epicardial electrophysiology.

**Results:** Visual inspection of reconstructed epicardial HCM maps revealed isolated patches of late activation time (AT), prolonged activation-recovery intervals (ARIs), as well as reversal of apico-basal trends in T-wave inversion and ARI compared to controls (*p* < 0.005 for all). AT and ARI were compared between groups. The pooled HCM group had longer mean AT (60.1 ms vs. 52.2 ms, *p* < 0.001), activation dispersion (55.2 ms vs. 48.6 ms, *p* = 0.026), and mean ARI (227 ms vs. 217 ms, *p* = 0.016) than structurally normal heart controls. HCM ventricular arrhythmia survivors could be differentiated from HCM patients without a personal history of life-threatening arrhythmia by longer mean AT (63.2 ms vs. 57.4 ms, *p* = 0.007), steeper activation gradients (0.45 ms/mm vs. 0.36 ms/mm, *p* = 0.011), and longer mean ARI (234.0 ms vs. 221.4 ms, *p* = 0.026). A logistic regression model including whole heart mean activation time and activation recovery interval could identify ventricular arrhythmia survivors from the HCM cohort, producing a C statistic of 0.76 (95% confidence interval 0.72–0.81), with an optimal sensitivity of 78.6% and a specificity of 79.8%.

**Discussion:** The HCM epicardial electrotype is characterized by delayed, dispersed conduction and prolonged, dispersed activation-recovery intervals. Combination of electrophysiologic measures with logistic regression can improve differentiation over single variables. Future studies could test such models prospectively for risk stratification of sudden death due to HCM.

## 1 Introduction

Hypertrophic cardiomyopathy (HCM) is a structural ventricular abnormality. Non-sustained ventricular tachycardia (NSVT) on 24-h ECG is the only electrophysiological marker backed by current guidelines, yet it has the highest hazard ratio among conventional risk factors (O'Mahony et al., 2014; Heart Rhythm et al., 2011).

Increased myocyte size, fibrosis, and myocyte disarray contribute to slow and discontinuous conduction (Roberts and Sigwart, 2005). Invasive paced fractionation, a marker of this discontinuous conduction, has already been shown to be an effective risk stratifier (Saumarez et al., 2008). Longer QRS duration, longer corrected QTc, and more complex T waves were found in HCM subjects than among controls (Barletta et al., 2004). ECG imaging (ECGi) has been used to describe greater activation dispersion dispersion in HCM patients with sustained ventricular arrhythmia or cardiac arrest than in controls, but exercise, repolarization, and HCM patients without potentially life threatening arrhythmia have not been examined (Perez-Alday et al., 2020).

To bridge these gaps in our knowledge, we tested two hypotheses using exercise testing and ECG imaging. First, HCM patients can be differentiated from controls using epicardial electrophysiological measures. Second, VF/VT survivors are differentiable within the HCM cohort from those without a personal history of life-threatening arrhythmia.

## 2 Materials and methods

Ethical approval was granted by the UK Health Research Authority and the Fulham Research Ethics Committee (London, United Kingdom) under references 14/LO/1318 and 17/LO/1660, respectively.

### 2.1 Patient selection

Sixty-nine subjects were recruited:

1. Seventeen survivors of ventricular fibrillation (VF) or sustained ventricular tachycardia (VT) and hemodynamic compromise with HCM (‘HCM VF’).
2. Twenty patients with HCM without a previous history of VF or sustained VT (“HCM controls”).
3. Ten survivors of VF in the context of single-vessel total occlusion and ST-elevation, or critical triple vessel disease. All patients had full revascularization, recovery of left ventricular function by echocardiographic criteria, and return to full exercise capacity and asymptomatic status for >1 year (“IHD VF controls”).
4. Eleven patients undergoing ablation of benign ventricular ectopy (“VE controls”) with ECGi guidance. They had normal echocardiography and/or MRI, no family history of cardiac electrical disease, and no symptoms of cardiac ischemia. Recruitment and testing took place prior to ablation.
5. Eleven asymptomatic relatives of patients with Brugada syndrome (“BrS relative controls”), proven not to have the same condition by a negative ajmaline challenge reaching a dose endpoint of 1 mg/kg (up to 120 mg total dose).

The European Society of Cardiology (ESC) HCM-SCD risk scores (O'Mahony et al., 2014) were calculated at the time of recruitment, based on index visit information, to simulate reviewing a patient for a “primary prevention” device.

### 2.2 Exercise ECGi testing and epicardial mapping

**FIGURE 1:**
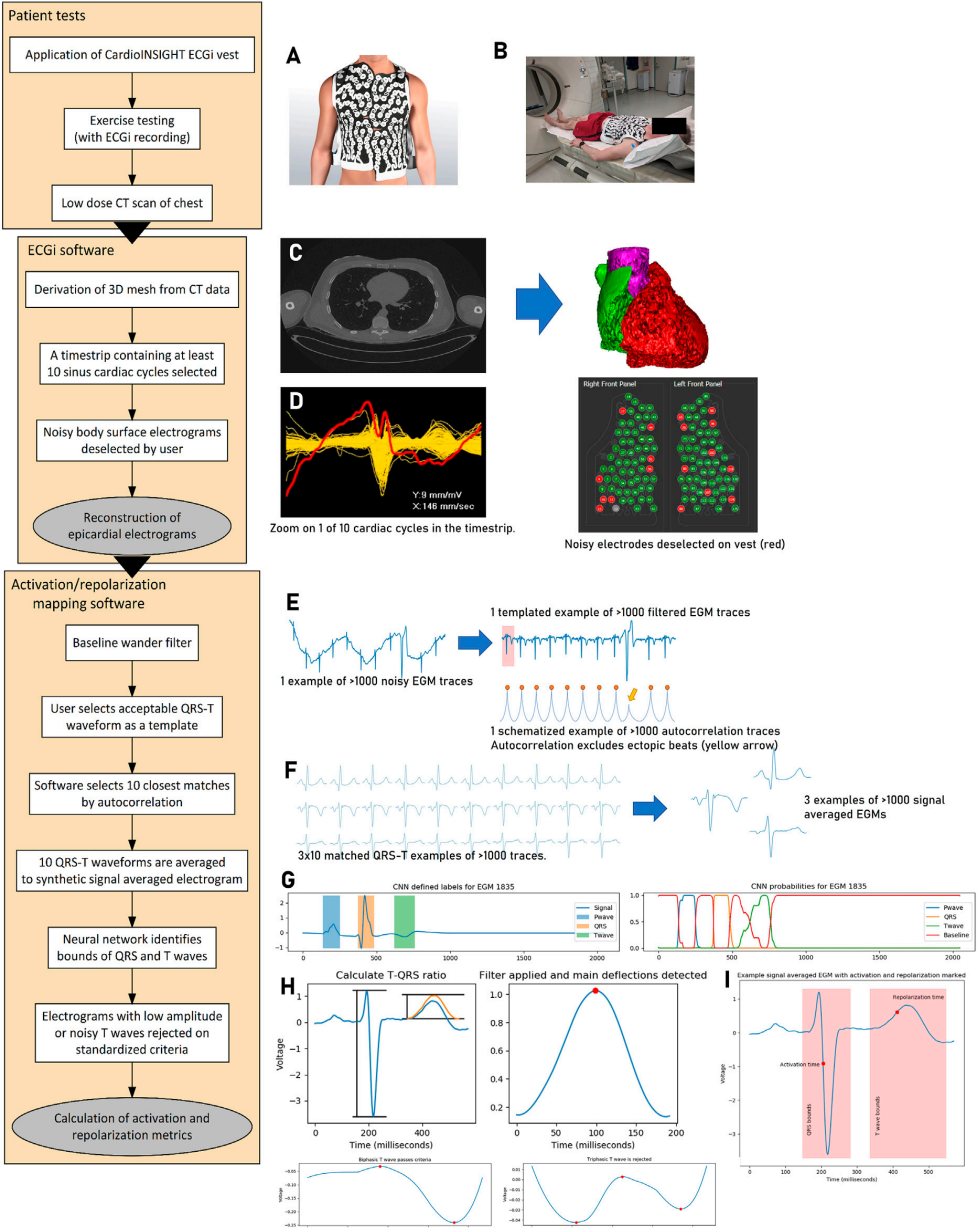
Activation–repolarization mapping process. **(A)** The 252-electrode sensor vest is applied to the patient undergoing maximal Bruce protocol exercise. Recordings are made during 10 min of supine recovery, which is followed by non-contrast CT scan of the chest **(B)**. The CT scan is segmented (**(C)**, left) into a 3D mesh (**(C)**, right). A time strip containing 10 cardiac cycles from the recording is selected for analysis, and body surface signals from the vest too noisy for analysis are identified (**(D)**, left) and removed from the vest recording (**(D)**, right). Epicardial electrograms are reconstructed and extracted to our custom software. In this mapping software, reconstructed electrograms (cf. the body surface signals from step **(D)** are filtered for baseline wander (**(E)**, left). The user selects a template QRS-T complex, and the software uses autocorrelation to search for the most similar 10 regions of interest (E, right). These 10 matched regions of interest containing the QRS complexes are signal averaged to a synthetic EGM per epicardial location **(F)**. A pretrained neural network identifies the bounds of the QRS complex and the T wave for further processing (**(G)**, left). It does this by working out the probability that a given time point is within a P wave, QRS complex, T wave, or the baseline based on its value and the values of its neighbors (**(G)**, right). EGMs with low amplitude relative to QRS or T waves with three or more deflections are rejected (indicative of poor interpretability, **(H)**. Following this, local activation and repolarization times can be calculated **(I)**. Electrocardiographic imaging, ECGi; computerized tomography, CT; three-dimensional, 3D; electrogram, EGM; convolutional neural network, CNN.

The ECGi mapping process is shown in Figure 1, and further details are provided in the Supplement. Beta blockers were avoided 48 h prior to testing. Volunteers wore a 252-electrode CardioINSIGHT™ vest and a heart rate monitor. Volunteers exercised to peak effort using the treadmill Bruce Protocol and then underwent 10 min of supine recording and computerized chest tomography (CT).

Epicardial EGMs were calculated using the CardioINSIGHT™ software. We designed custom software to filter, signal average, automatically segment electrograms, and exclude noise. Local activation time (AT) was defined from QRS start to the minimum dV/dt (steepest negative point) of the QRS complex, and local activation-recovery intervals (ARIs) were defined as AT to the maximum dV/dt (steepest positive point) of the T wave (Wyatt method (Wyatt et al., 1981)). Gradients were measured between electrograms within a 5-mm boundary.

Three domains were defined for analysis:

1. The mean of activation or ARI was used to describe overall *conduction delay* or as a marker of *abnormally prolonged repolarization.*
2. The central 95% range of times was used to describe *dispersion.*
3. The mean gradient of activation and ARI times in space was used to compare the *steepness of gradients.*

### 2.3 Logistic regression for the description of the arrhythmogenic substrate in HCM

Logistic models were used to determine the contribution of significant differentiators. Qualifying measures (univariate *p* < 0.05) were scaled and collinear measures rejected before model fitting using Newton’s method. Backward stepwise selection was used to reject variables with *p* > 0.15 (Hosmer et al., 2013; Chowdhury and Turin, 2020). The predicted probability of an observation falling into the HCM VF group was compared for the true HCM VF group and the HCM patient group without a history of previous arrhythmia. To determine the ability of these logistic models to predict whether a patient was in the HCM or HCM VF group in a wider population, k-fold cross-validation was performed. Five folds were chosen based on the size of the dataset.

### 2.4 Statistical analysis

Comparisons across more than two groups were carried out using the Kruskal–Wallis test as measures were not distributed in a Gaussian fashion. Intergroup comparisons were made using the Wilcoxon rank-sum test. Proportions were tested by the T test, and where appropriate, the effect size was estimated by Cohen’s h. Significance was defined as *p* < 0.05. Data were analyzed in R v4.0.3 and Python v3.7.

## 3 Results

### 3.1 Patient characteristics, surface measures, and electrogram deselection

Table 1, 2 summarize patient characteristics and surface ECG markers, split into planned comparisons for this investigation. Table 1 concerns the pooled HCM cohort (HCM VF survivors and other HCM patients without VF) versus the pooled normal heart controls (recovered IHD VF, VE, and BrS relatives). Table 2 compares HCM patients with VF to HCM controls without VF.

**TABLE 1:** Characteristics of volunteers undergoing electrocardiographic imaging exercise testing. HCM patients with and without VF/VT were pooled to provide a comparison with normal heart controls. For separate analysis, see Table 2. The normal heart controls included Brugada syndrome relatives screened for heart disease and confirmed normal, asymptomatic, revascularized ischemic VF patients with full recovery of ventricular function and patients undergoing ablation for benign ventricular ectopy. Peak and recovery phase heart rates were those when the signal was clean enough for measurement using the electrocardiographic imaging system. Measures are presented as mean ± standard deviation. Bold *p*-values are statistically significant. Hypertrophic cardiomyopathy, HCM, ventricular fibrillation/tachycardia; VF/VT.

Parameter	Pooled HCM	Normal heart controls	*p*-value
Count	37	32	-
Males (proportion)	0.76	0.68	0.46
Age (years, mean)	49.5 ± 14.4	48.2 ± 13.6	0.58
Peak phase heart rate (bpm, mean)	132.4 ± 24.7	128.6 ± 23.3	0.56
Recovery phase heart rate (bpm, mean)	79.0 ± 15.8	90.5 ± 18.1	**0.006**
Peak phase QRS duration (ms, mean)	90.9 ± 22	91.7 ± 13.8	0.41
Recovery phase QRS duration (ms, mean)	103.1 ± 17.2	103.1 ± 13.6	0.89
Peak phase-corrected QT interval (ms, mean)	355.8 ± 38.6	342.3 ± 25.4	0.17
Recovery phase-corrected QT interval (ms, mean)	412.7 ± 32.9	395.1 ± 31.2	**0.04**

**TABLE 2:** Characteristics of volunteers with HCM undergoing electrocardiographic imaging exercise testing. Peak and recovery phase heart rates were those when the signal was clean enough for measurement using the electrocardiographic imaging system. Measures are presented as mean ± standard deviation. Bold *p*-values are statistically significant. Hypertrophic cardiomyopathy, HCM; HCM ventricular fibrillation or hemodynamically unstable sustained ventricular tachycardia survivor, HCM VF; European Society of Cardiology, ESC; ventricular tachycardia, VT.

Parameter	HCM VF	HCM	*p*-value
Count	17	20	-
Males (proportion)	0.76	0.76	0.91
Age (years, mean)	45.5 ± 14.8	52.0 ± 13.9	0.09
Mean ESC score (5-year risk, %)	5.90 ± 5.5	2.85 ± 2.3	**0.023**
Syncope (proportion [count])	0.17 [3]	0.05 [1]	0.20
Max left ventricular hypertrophy (mm)	19.4 ± 4.6	18.6 ± 3.7	0.40
Left atrial size (mm)	40.3 ± 7.3	39.5 ± 4.6	0.82
Left ventricle outflow gradient (mmHg)	26.8 ± 46.1	23 ± 26.5	0.54
Non-sustained VT history (proportion [count])	0.53 [9]	0.29 [6]	0.13
Early familial sudden death (proportion [count])	0.35 [6]	0.14 [3]	0.14
Peak phase heart rate (bpm, mean)	125.3 ± 25.6	138.4 ± 22.9	0.11
Recovery phase heart rate (bpm, mean)	70.3 ± 14.3	86.5 ± 13.2	**0.001**
Peak phase QRS duration (ms, mean)	94.2 ± 17.5	88.2 ± 25.3	0.13
Recovery phase QRS duration (ms, mean)	104.7 ± 15.6	101.7 ± 18.8	0.71
Peak phase-corrected QT interval (ms, mean)	361.3 ± 30.2	351.2 ± 44.8	0.17
Recovery phase-corrected QT interval (ms, mean)	427.4 ± 30	400.1 ± 30.6	**0.01**

All patients reached 85% of age-predicted maximal heart rate during peak exertion. The HCM VF or hemodynamically unstable VT group name is simplified to “HCM VF” hereon. Only two patients in this group had sustained VT, and only one of these had an apical aneurysm. One patient from the HCM VF group was excluded as their implantable device began back-up pacing during recovery.

The pooled HCM and normal heart control groups were similar in sex and age. The pooled HCM group had lower recovery phase heart rates (mean ± SD 79.0 ± 15.8 bpm vs. 90.5 ± 18.1 bpm *p* = 0.006) and longer corrected QT (mean ± SD 412.7 ± 32.9 ms vs. 395.1 ± 31.2 ms, *p* = 0.04). These differences were mainly driven by the HCM VF group: in pairwise analysis, HCM VF patients had lower heart rates than HCM, BrS relative and VE counterparts (vs. HCM, *p* = 0.0014; vs. BrS relative, *p* = 0.0001; vs. VE, *p* = 0.0028) and longer QTc than any group, except VE (vs. BrS relatives, *p* = 0.00014; vs. IHD VF, *p* = 0.002; vs. HCM, *p* = 0.01).

The HCM VF and HCM control groups were similar in sex and age. The HCM VF group had higher ESC risk scores than the HCM group, although 8 of 17 HCM VF patients had a score <4%/5-year risk and 11 had a score <6%/5-year risk. None of the subcomponents of the ESC score significantly differentiated VF survivors from asymptomatic HCM patients. The HCM and HCM VF groups had similar hypertrophy distributions (4/20 vs. 2/17 non-septal, *p* = 0.8, Cohen’s h = 0.22). HCM VF patients had lower recovery phase heart rates (70.3 ± 14.3 vs. 86.5 ± 13.2 bpm, *p* = 0.001) and longer corrected QT (427.4 ± 30 vs. 400.1 ± 30.6 ms, *p* = 0.01). Peak phase heart rates and QTc were similar. QRS durations were same between the groups in either phase.

Surface measurements could not be used as a substitute for reconstructed epicardial estimates as they were poorly correlated. Mean AT and QRS duration shared a Spearman’s R^2^ = 0.117, *p* = 0.018; mean-corrected ARI and QTc had a Spearman’s R^2^ = 0.204, *p* = 0.001.

During the process, both body surface electrodes (manual) and epicardial EGMs (automated) were selected if too noisy for analysis. The proportion of selected body surface electrodes was 62.1% ± 12.2% out of a possible 252, and there was no influence of greater deselection with either mean AT or corrected ARI (Spearman’s R^2^ = 0, 0.08; *p* = 1, 0.04 respectively); 9.1% ± 11.5% of epicardial EGMs were deselected, and there was no influence of greater deselection on AT or ARI (Spearman’s R^2^ = 0.032, 0.144; *p* = 0.228, 0.008, respectively).

### 3.2 Electrophysiological phenotype of hypertrophic cardiomyopathy

**FIGURE 2:**
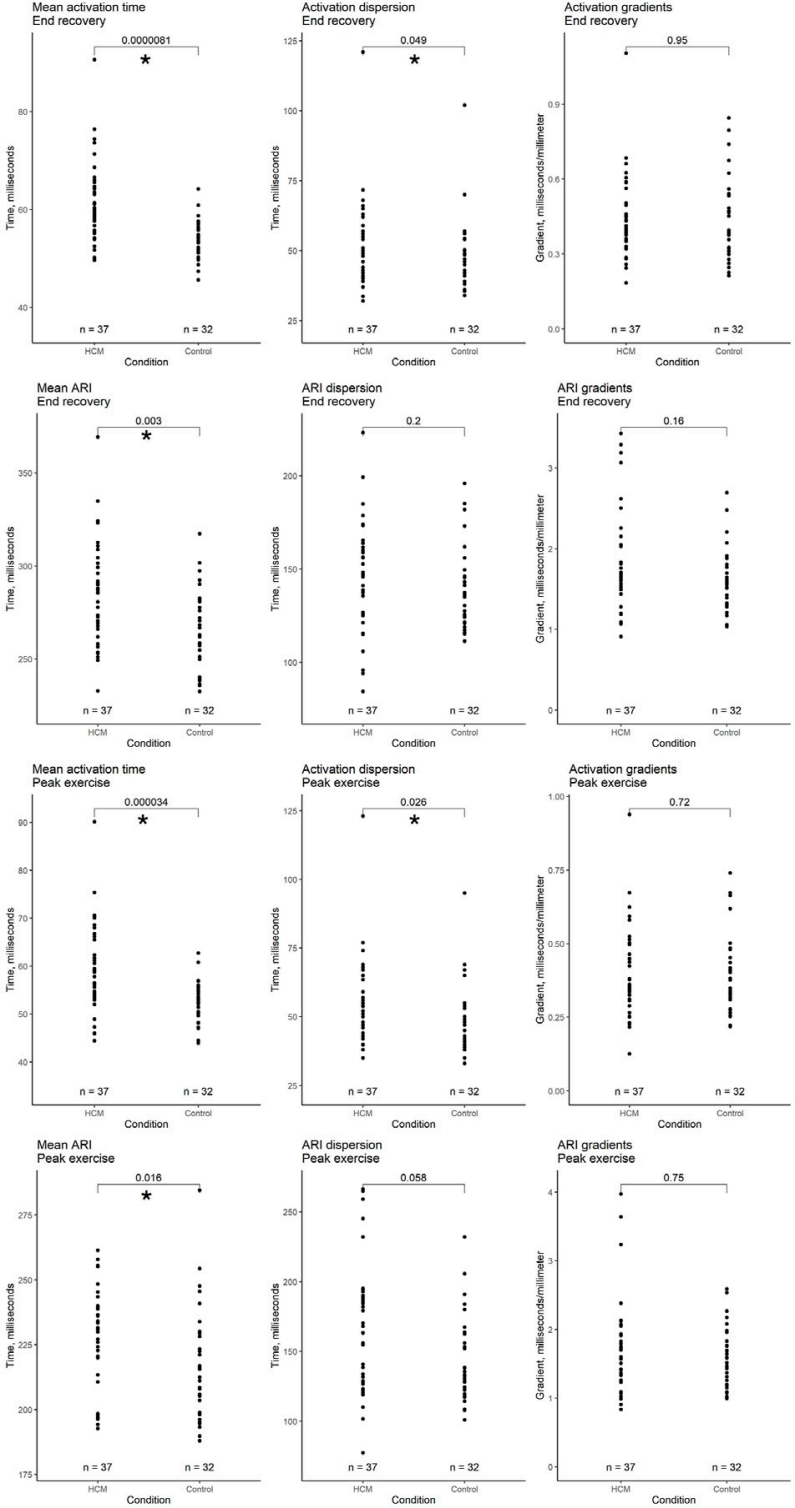
Comparison of whole heart activation and repolarization metrics immediately after peak exercise and in end recovery between a pooled hypertrophic cardiomyopathy cohort (HCM + HCM VF groups) and a pooled selection of structurally normal heart controls. Local activation time (LAT) was defined as the onset of the first epicardial QRS complex to the steepest negative slope of the electrogram–QRS complex. Local repolarization time (LRT) was defined as the onset of the first epicardial QRS complex to the steepest positive slope of the electrogram-T wave. Activation recovery interval (ARI) is the difference between LAT and LRT. Mean time is the average of all LAT/ARI across the heart. Dispersion is the central 95% range of LAT/ARI across the heart. Gradient is the whole heart mean rate of range in LAT/ARI over a 5 mm search distance around each epicardial location. An asterisk (*) denotes *p*-value < 0.05.

To determine the electrophysiological features of HCM, we compared the pooled HCM group with normal heart controls. Figure 2 graphically summarizes the findings.

#### 3.2.1 Peak exercise

Following exercise, mean AT was longer in the pooled HCM group (mean ± SD 60.1 ± 9.4 ms vs. 52.2 ± 4.3 ms, respectively, *p* < 0.001). Activation dispersion was longer in HCM patients (55.2 ± 16 ms vs. 48.6 ± 12.1 ms, respectively, *p* = 0.026). Activation gradients were similar (*p* = 0.72).

The pooled HCM group had longer mean ARI than normal heart controls (mean 227 ms vs. 217 ms, *p* = 0.016). ARI dispersion and gradients were similar (*p* = 0.058, 0.75).

#### 3.2.2 End recovery

Following recovery, mean AT was longer in the pooled HCM group than in structurally normal heart controls (mean ± SD 61.3 ± 8.4 ms vs. 54.1 ± 3.9 ms, respectively, *p* < 0.001), as was activation dispersion (53.2 ± 15.2 ms vs. 48.2 ± 12.4 ms, *p* = 0.049). Activation gradients were similar (*p* = 0.95).

The pooled HCM group had longer mean ARI than normal heart controls (mean ± SD 286 ± 28 ms vs. 265 ± 22 ms, respectively, *p* = 0.003). ARI dispersion and gradients were similar (across groups *p* = 0.2, 0.38).

### 3.3 Visual representations of automated measurements

**FIGURE 3:**
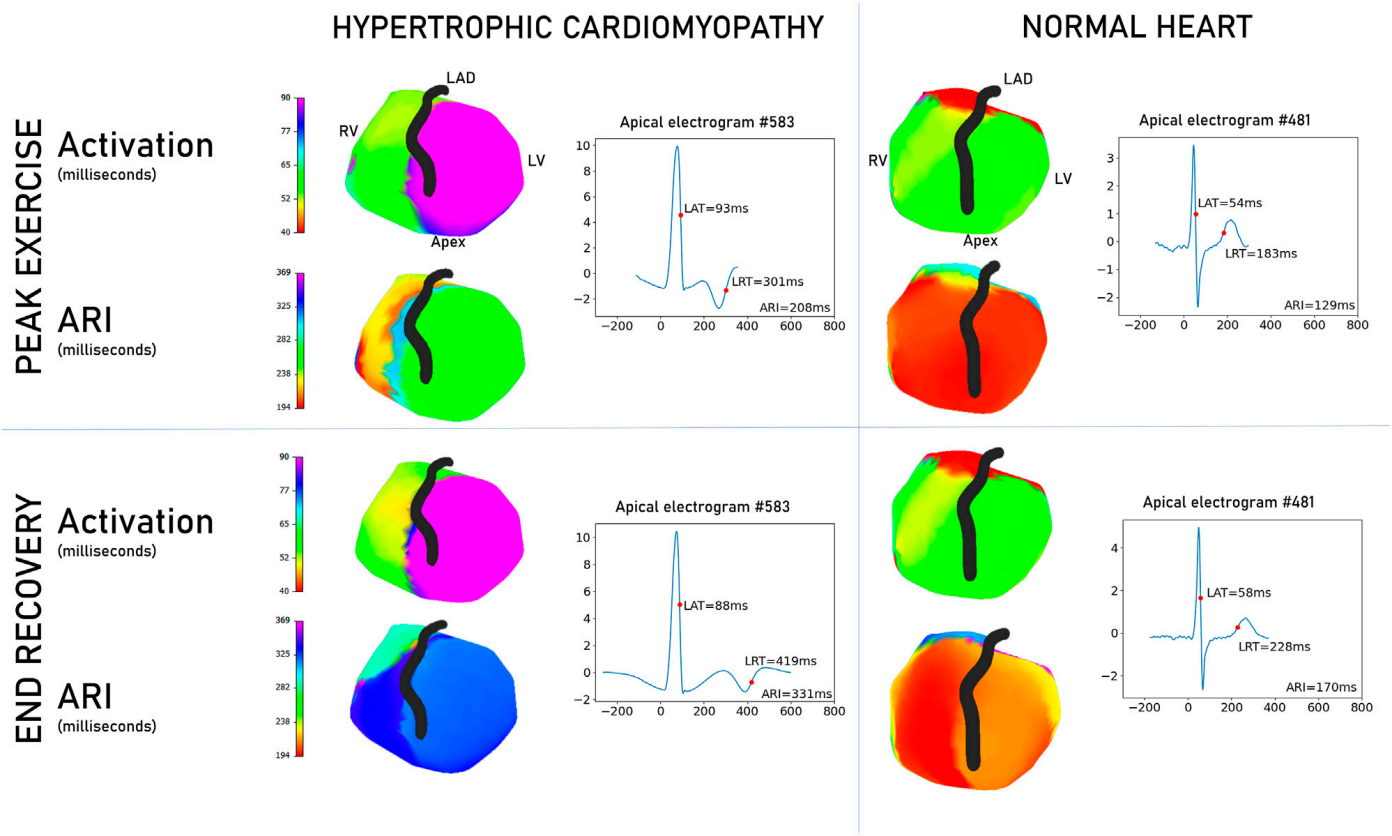
Comparison of non-invasive epicardial maps between a patient with hypertrophic cardiomyopathy and an asymptomatic, unaffected Brugada syndrome counterpart. Scales are matched for activation and repolarization separately to aid comparison. Examples are selected to illustrate the differences seen in the overall cohort. In activation, the hypertrophic cardiomyopathy heart (left panel) has delayed conduction and repolarization compared to the normal heart (right panel). Apical electrograms are displayed for both hearts, with the HCM heart exhibiting T-wave inversion. Graphs are voltage/time, where zero time is the global QRS start. Right ventricle, RV; left ventricle, LV; left anterior descending artery, LAD; local activation time, LAT; local repolarization time, LRT; activation recovery interval, ARI.

Figure 3 shows epicardial maps of a patient with HCM and of a Brugada syndrome relative (normal heart control). The HCM patient’s heart has delayed conduction and slower repolarization than the normal heart. Lines of steep activation or repolarization change can be observed on the HCM epicardium. HCM electrograms show abnormal T-wave inversion, not seen in the normal heart subject.

**FIGURE 4:**
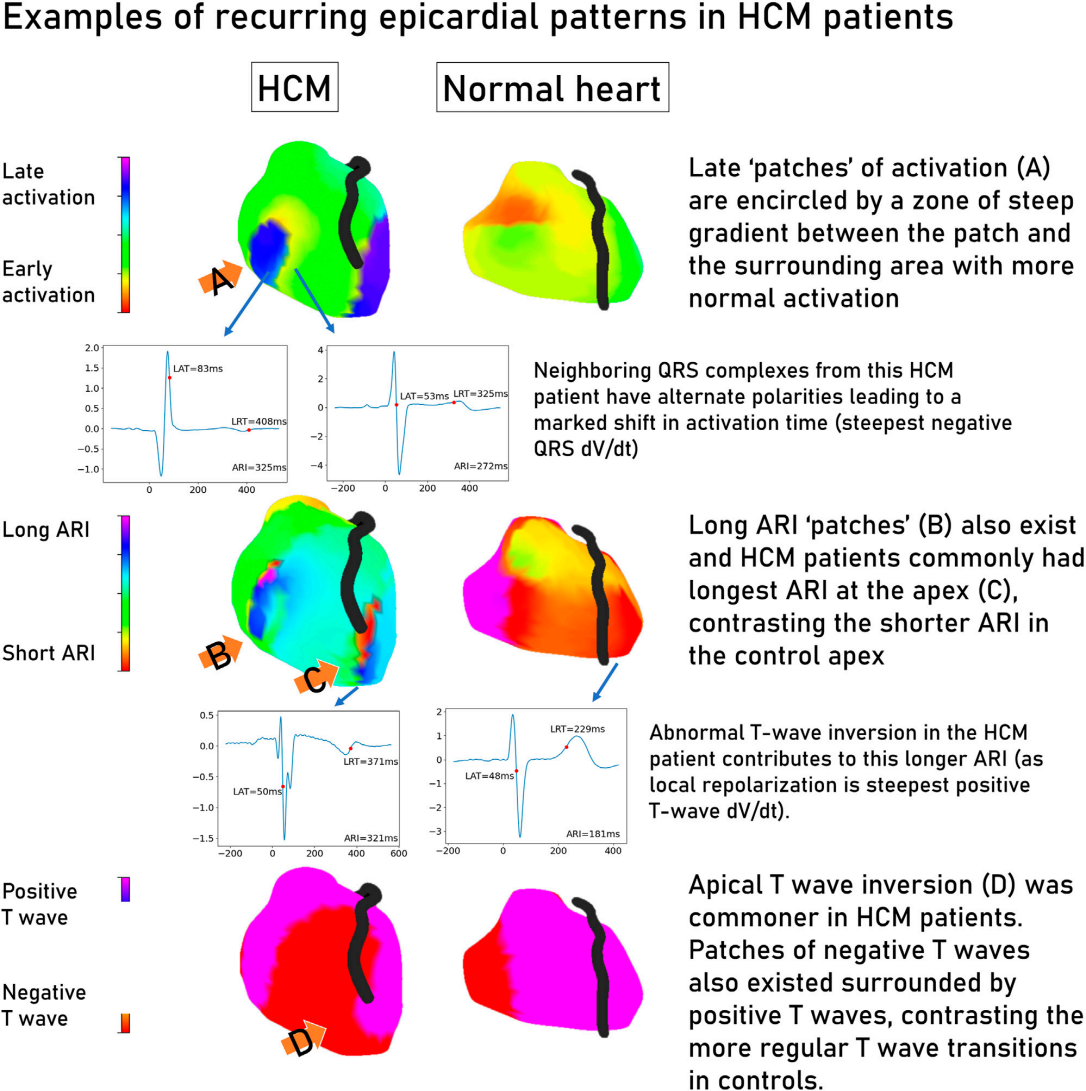
Common patterns seen in electrocardiographic imaging maps of hypertrophic cardiomyopathy patients contrasted with a structurally normal heart. Representative electrograms are linked to their location on the epicardial shell by an arrow. Graphs are voltage/time, where zero time is the global QRS start. Hypertrophic cardiomyopathy, HCM; change in voltage over time, dV/dt; local activation time, LAT; local repolarization time, LRT; activation recovery interval, ARI.

Figure 4 shows some common epicardial patterns noted in HCM patients. Late, isolated patches of activation surrounded by a high activation gradient were most common in HCM patients (72.9% vs. 31.2% of controls, *p* < 0.001) and could be found anywhere on the epicardial surface. Relatively prolonged ARI at the apex was commoner in HCM (67.5% vs. 31.5% controls, *p* = 0.002), as were negative T waves at the apex (HCM 75.6% vs. 40.6% controls, *p* = 0.003). Isolated patches of negative T waves in areas of otherwise predominantly positive T waves were also more common in HCM patients (59.4% vs. 12.5% controls, *p* < 0.001).

### 3.4 Electrophysiological phenotype of ventricular fibrillation survivors with HCM

**FIGURE 5:**
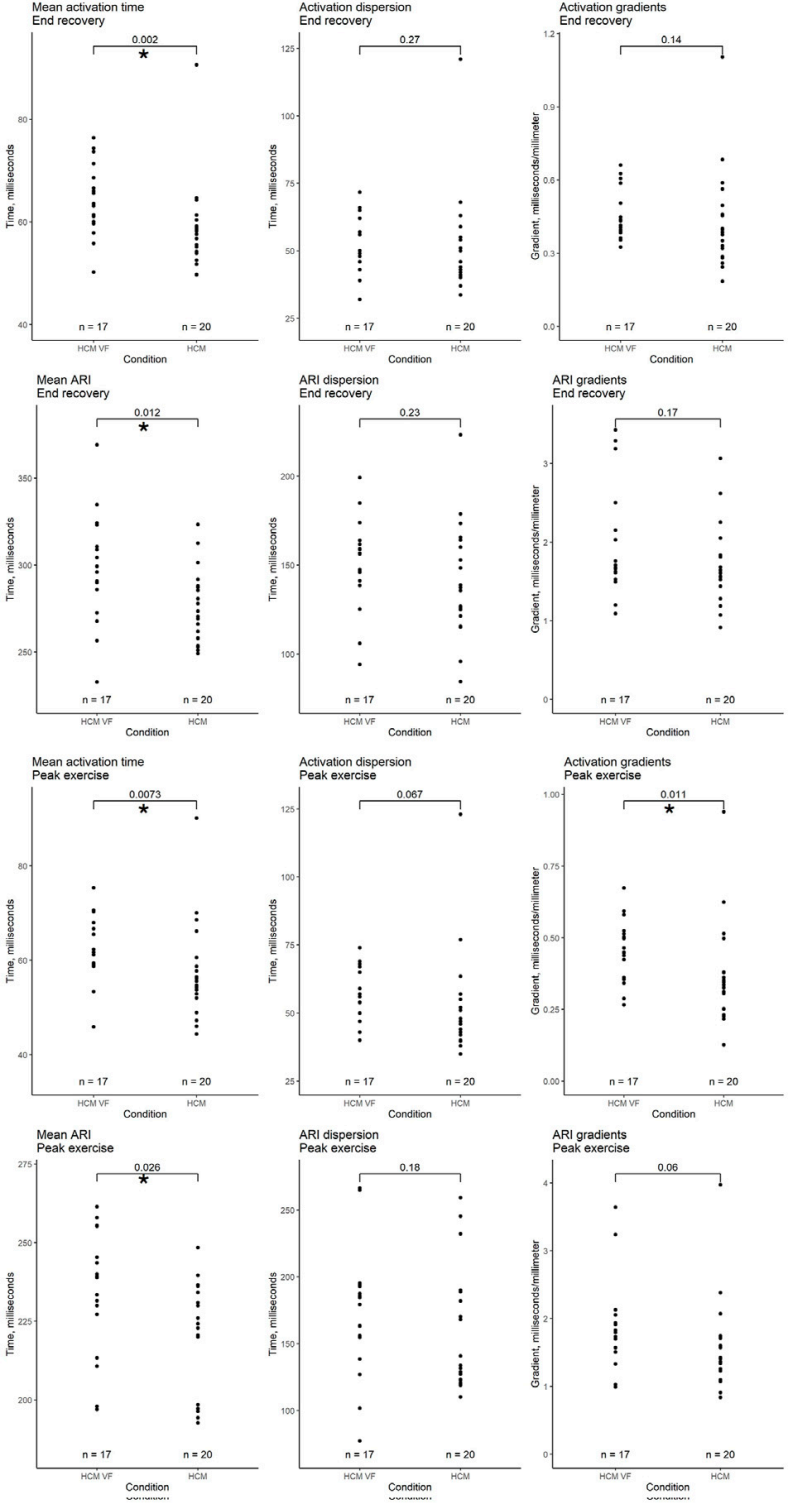
Comparison of whole heart activation and repolarization metrics immediately after peak exercise and in end recovery between hypertrophic cardiomyopathy (HCM) patients without a personal arrhythmic history and VF or hemodynamically unstable VT survivors (HCM VF). Local activation time (LAT) was defined as the onset of the first epicardial–QRS complex to the steepest negative slope of the electrogram–QRS complex. Local repolarization time (LRT) was defined as the onset of the first epicardial–QRS complex to the steepest positive slope of the electrogram-T wave. Activation recovery interval (ARI) is the difference between LAT and LRT. Mean time is the average of all LAT/ARI across the heart. Dispersion is the central 95% range of LAT/ARI across the heart. Gradient is the whole heart mean rate of range in LAT/ARI over a 5 mm search distance around each epicardial location. An asterisk (*) denotes a *p*-value < 0.05.

We compared electrophysiological features of HCM VF survivors with those of HCM patients without a potentially lethal ventricular arrhythmia. Figure 5 summarizes the significant variables. All variables (including non-significant) are graphed in the Supplement.

#### 3.4.1 Peak exercise

Following peak exercise, HCM VF survivors had longer mean AT (mean ± SD 63.2 ± 7.2 ms vs. 57.4 ± 10.3 ms, *p* = 0.007). Activation dispersion trended toward being longer in HCM VF patients (mean ± SD 56.9 ± 9.5 ms vs. 53.6 ± 20.1 ms, *p* = 0.067). Activation gradients were steeper in HCM VF patients (mean ± SD 0.45 ± 0.11 ms/mm vs. 0.36 ± 0.18 ms/mm, *p* = 0.011).

HCM VF patients had a longer mean ARI (mean ± SD 234.0 ± 19.8 ms vs. 221.4 ± 16.8 ms, *p* = 0.026). ARI dispersion was similar between groups, but ARI gradients tended to be steeper in HCM VF patients than in HCM counterparts (mean ± SD 1.89 ± 0.67 ms/mm vs. 1.58 ± 0.67 ms/mm, *p* = 0.06).

#### 3.4.2 End recovery

Following recovery, HCM VF survivors had a longer mean AT (mean ± SD 64.4 ± 7.0 ms vs. 58.6 ± 8.6 ms, *p* = 0.002). ARIs were longer in HCM VF patients (mean ± SD 298 ± 31.5 ms vs. 275.5 ± 20.9 ms, *p* = 0.012). Neither AT nor ARI dispersion and gradients were significant differentiators.

### 3.5 Ventricular conduction stability

We previously described ventricular conduction stability (V-CoS) as a tool to quantify activation heterogeneity in response to exercise (Shun-Shin et al., 2019). The pooled HCM cohort had significantly less preserved activation patterns in response to exercise compared to the normal heart controls (mean ± SD 96.5% ± 3.9% vs. 98.9% ± 0.8% V-CoS, respectively, *p* = 0.015). HCM VF patients were not significantly different from HCM controls (*p* = 0.89).

### 3.6 Logistic regression for the description of the arrhythmogenic substrate in HCM

Mean AT and ARI in recovery and mean AT, activation gradients, and mean ARI following peak exercise were significantly higher in the HCM VF patient group. Mean AT in exercise and recovery strongly correlated (Pearson R = 0.93, *p* > 0.001), so mean AT in exercise was excluded to reduce collinearity. The remaining four variables had Pearson correlations from 0.21 to 0.72 (Supplementary Material).

An initial 4-variable model (mean AT and mean ARI in recovery, mean ARI, and activation gradients following peak exercise) was built to differentiate HCM VF patients from HCM patients without VF. This 4-variable model achieved a log-likelihood ratio (LLR) *p*-value of 0.04 (lower is better) with a sensitivity of 0.82 and a specificity of 0.8. Individual coefficients however were non-significant (*p* = 0.14–0.82, see Supplementary Material).

**FIGURE 6:**
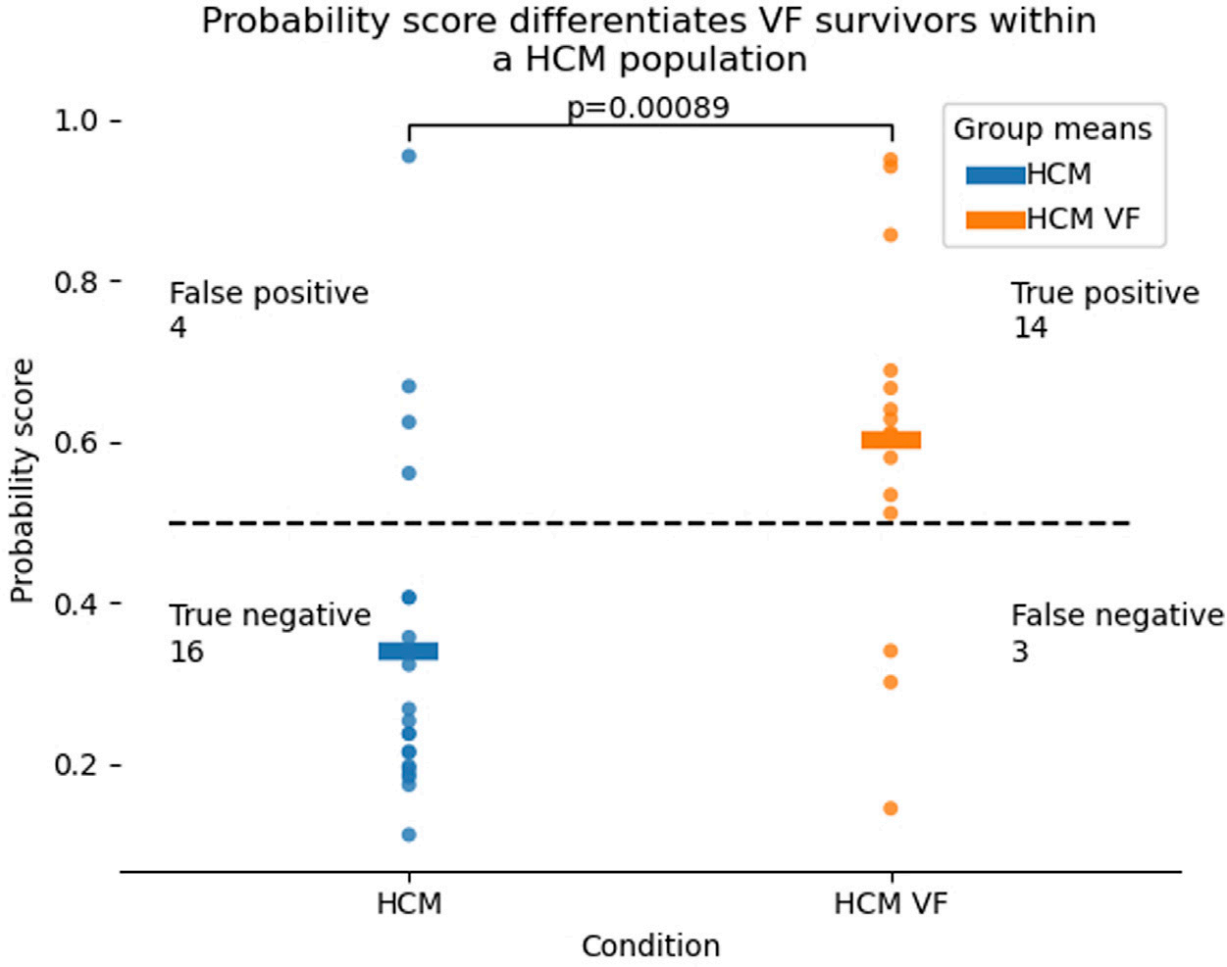
Probability distributions for HCM VF or unstable VT survivors as well as HCM patients without a personal history of life-threatening arrhythmia, produced by a 2-variable logistic model of mean activation time and mean activation recovery interval at rest. Higher probability scores refer to the chance that the patient in question falls in the HCM VF group. The dotted line represents a probability of 0.5. Correct classification was defined as *p* > 0.5 for HCM VF and *p* < 0.5 for HCM, although this threshold can be defined differently by the clinician. Hypertrophic cardiomyopathy, HCM; ventricular fibrillation, VF; ventricular tachycardia, VT.

Stepwise exclusion of the least significant coefficient was performed until all coefficients reached the prespecified stop criterion (*p* > 0.15). The resulting 2-variable model of mean AT and ARI in full recovery reached an LLR *p* = 0.008. This 2-variable model differentiated the HCM VF and HCM groups better than any single variable from our panel (Figure 6), similarly to the 4-variable model, and better than a model consisting of only surface ECG measures which could only manage a balanced accuracy of 0.62 (Supplementary Material).

Mean AT had an odds ratio of 1.1 for being in the HCM VF group (95% confidence intervals 0.98–1.23) per millisecond increase, and the mean ARI had an odds ratio of 1.03 (95% confidence intervals 1.00–1.06) per millisecond increase.

### 3.7 Ability of a multiple logistic model to predict in unseen data

To determine the ability of these logistic models to predict whether a patient was in the HCM or HCM VF group in a wider population, k-fold cross-validation was performed.

**FIGURE 7:**
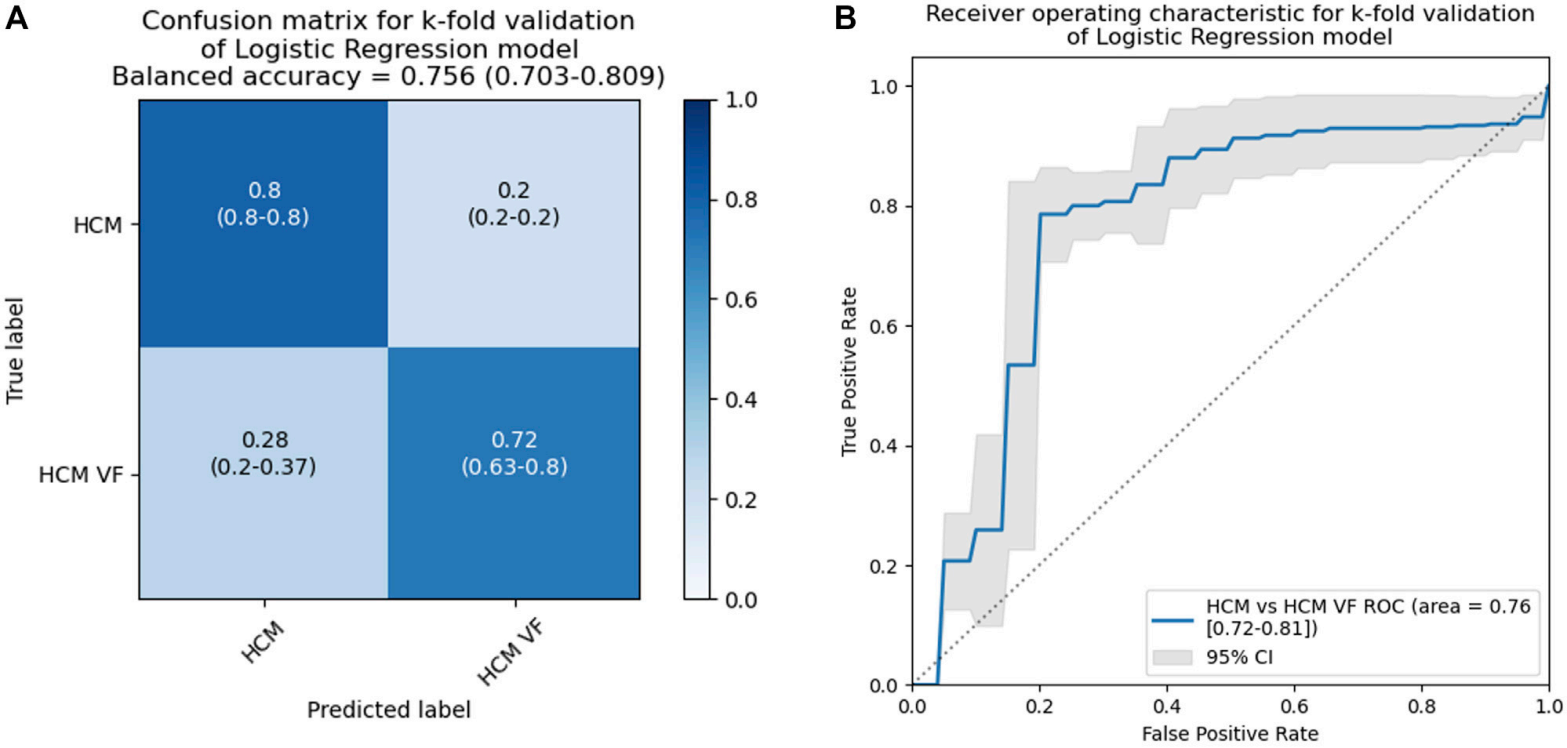
Results for a k-fold cross-validation of 2-variable logistic models including mean activation times and ARI at rest in patients with HCM without a personal history of life-threatening arrhythmia and HCM VF or hemodynamically unstable VT survivors. This analysis simulates unseen data to provide a more reliable estimate of how a model with the same input variables will generalize. The classification threshold was set to *p* = 0.5, and the dataset was split into five folds. HCM, hypertrophic cardiomyopathy; VF, ventricular fibrillation; VT, ventricular tachycardia.

A five-fold validation was performed. The balanced accuracy of the 2-variable models was 0.75 (95% confidence intervals [CI]: 0.70–0.80, Figure 7A). Individual accuracy for conditions was 0.80 for HCM (95% CI: 0.8–0.8) and 0.72 for HCM VF (95% CI: 0.63–0.8). The 4-variable models would have only produced accuracies of 0.8 and 0.62 for HCM and HCM VF, respectively. The receiver operating characteristic (ROC) area under the curve was 0.76 (95% CI: 0.72–0.81, Figure 7B). The aggregated models could achieve a Youden’s sensitivity of 78.6% with a specificity of 79.8% for identifying a patient from the HCM VF group.

## 4 Discussion

In this study, we used non-invasive ECGi to show differences in activation and repolarization between patients with HCM and those with structurally normal hearts. Activation and repolarization changes also differentiate HCM VF patients from HCM patients without VF, and logistic regression of these markers can improve the identification rate of HCM VF survivors from the HCM cohort.

### 4.1 Traditional risk markers and surface ECG characteristics

Approximately half of the HCM VF event patients would not have had an implantable cardioverter defibrillator (ICD) recommended based on their ESC score prior to the arrhythmic event, corroborating the observations of previous publications examining ESC scores in arrest survivors (Maron et al., 2015; Leong et al., 2018). Prolonged QT has not been included in risk stratification guidelines (Gray et al., 2013), but has been noted in arrhythmia-prone patients and was also observed in the HCM VF patients in our study. Although a significant difference was found between groups, as Figure 2 demonstrates low *p*-values can hide such overlap that defining a cut-off for risk is challenging. In the Supplement, we made a multivariate model of surface ECG characteristics only to test whether ECGi showed any incremental benefit—the surface ECG only model was outperformed by the ECGi model.

ECG imaging provides different information to our standard ECG intervals—QRS durations were similar between groups, QRS duration did not correlate well with AT, and AT was a strong differentiator of the groups. An incremental benefit may be had from high-density body surface mapping over standard 12-lead ECG—this would need to be studied further in the future.

### 4.2 The HCM epicardial electrotype

Our study showed that electrical activation is delayed and takes longer to complete (dispersion) in HCM patients with higher gradients across the epicardium than the controls, and this is consistent with the observations of previous publications (Schumacher et al., 2005; Perez-Alday et al., 2020). We also show that abnormal repolarization by means of prolonged ARI exists independently from the activation changes (Supplement). Patterns of late patches of activation or prolonged repolarization, as well as apico-basal reversal of T-wave inversion and activation recovery time, were noted as general trends separating HCM and control; further work could determine the relationship to scar by co-registering with MRI data. This was not possible in the current cohort due to the number of non-MRI conditional devices preventing the acquisition of late gadolinium enhanced images.

Previous studies showed HCM patients had longer surface QT intervals and T peak-T end measurements than healthy controls without evidence of ventricular hypertrophy (Jalanko et al., 2018). These measures are correlates of mean and dispersion of epicardial ARI. Our study suggests that mean ARI is a stronger differentiator of HCM than ARI dispersion. The near-asymptotic nature of T-wave termini is also a challenge for reproducible measurement; a steepest positive strategy is more easily definable.

### 4.3 Understanding contributors to ventricular fibrillation in HCM using logistic regression

We showed that HCM VF survivors had longer mean AT, steeper activation gradients, and longer mean ARIs than HCM controls. Indirect corroboration of these findings comes from invasive studies measuring paced fractionated electrogram designed to detect the effects of myocyte disarray on intraventricular conduction (Saumarez et al., 1992). Our results show this conduction slowing non-invasively and as a continuous variable in patients who cannot be differentiated by QRS duration. QTc, a risk marker for appropriate shocks (Gray et al., 2013), combines both activation and repolarization; our epicardial study measures ARI, an accepted correlate of action potential duration (Haws and Lux, 1990). Thus, we found that ventricular repolarization is prolonged in HCM VF survivors independent of the activation pattern. Notably, some HCM VF survivors had overlapping electrical characteristics with both HCM patients without previous cardiac arrest and the control population. Disarray in these patients may be either minimal in these patients or may be causing abnormal conduction velocity by direction of impulse rather than scalar speed (Finocchiaro et al., 2021).

Our logistic regression model could be simplified down to two variables: mean AT in recovery and mean ARI in recovery. Odds ratios suggest that longer mean AT and ARI independently increase the risk of falling in the VF category. The independence and poor correlation of activation and repolarization measures in our model suggest that there is more than one mechanism for VF in HCM. Further study would be required to subtype HCM patients with activation predominant or repolarization predominant disease. Future therapies may be tailored by the findings of mapping studies.

### 4.4 The contribution of exercise to the arrhythmic substrate of HCM

We describe activation and repolarization abnormalities in HCM patients immediately following cessation of maximal exercise, which are persistent into recovery. This contrasts with the findings of previous studies demonstrating a dynamic response to elevated heart rates either from exercise or pacing (Saumarez et al., 2008; Leong et al., 2020). The V-CoS sub-analysis performed in this study shows that some exercise-induced changes can only be appreciated by a measure that appreciates electrogram location, even though simpler mean activation and repolarization times were superior differentiators of the arrhythmia survivors. Our findings may explain why the traditional view of HCM sudden death as an intense exercise-linked phenomenon is being challenged by more recent registry (Lampert et al., 2023).

### 4.5 Limitations

The small size of this pilot study predisposes it to type II error, and non-significant variables cannot be fully discounted from future study. The significance of measures such as activation gradients in exercise but not in recovery could be important in larger studies and explain the possible link between exercise and HCM death (Margey et al., 2011).

Correlation coefficients between invasive epicardial maps and ECGi reconstructions have been quoted between 0.03 and 0.86 (Cluitmans et al., 2017; Graham et al., 2018; Duchateau et al., 2019). However, all *in vivo* invasive mapping comparisons to date have suffered from difficulty co-localizing and timing against ECGi reconstructed points. Invasive contact maps by roving catheter are constructed on the assumption that multiple cardiac cycles are identical, while ECGi maps are collected in a single beat, allowing the discernment of beat-to-beat variations which cannot be captured by contact mapping. Spatial mismatch in these studies could reach 20 mm, which in our normal controls could lead to activation time mismatch of up to 10 ms. Graham et al. recognized this effect directly in their validation paper (Graham Adam et al., 2019).

Concordance with the (unknown) ground truth will be a problem for any ECGi-based project of this type. Notably in this paper, we did not perform invasive mapping on our patients; this has implications on elucidating the mechanisms of arrhythmia. On one hand, spatial differences are less interpretable from our maps than for a roving catheter map. To reduce the effect of spatiotemporal disagreement with invasive ground truth, we chose to signal average and use spatially agnostic statistics. Papers based on roving catheter maps will in turn not be able to assess exercise-induced or temporal change as well as a non-invasive, single beat strategy such as ECG imaging.

### 4.6 Conclusion

The HCM epicardial electrotype is characterized by delayed, dispersed conduction and prolonged, dispersed repolarization, often accentuated by exercise. These factors occur independently, reinforcing the view that hypertrophic cardiomyopathy is a heterogeneous disease. These parameters may be useful for risk stratification of sudden cardiac arrest, but larger prospective trials would be recommended to test the findings generated by this pilot study.

## Data Availability

The data that support the findings of this study are not publicly available due to their containing information that could compromise the privacy of research participants. The data are available on request from the corresponding author, PK.
