# Supplementary material for: The arrhythmic substrate of hypertrophic cardiomyopathy using ECG imaging
Source: Front Physiol. 2024 Aug 14;15:1428709. doi: 10.3389/fphys.2024.1428709 (PMC11350108; doi:10.3389/fphys.2024.1428709)
Supplement: Supplementary file 1 [file DataSheet1.pdf]

# Supplementary Material

## 1 Exercise ECGi testing and epicardial mapping

This text accompanies Figure 1 and the Methods section in the main paper.

The ECGi mapping process is graphically represented in Figure 1. Beta blockers were omitted 48 hours prior to testing. Each volunteer was fitted with the appropriately sized 252-electrode CardioInsight™ vest based on height and body shape, and a heart rate monitor. Volunteers exercised to peak effort (defined as exhaustion and exceeding 85% of predicted maximum heart rate) using the treadmill Bruce Protocol, then underwent 10 minutes of recording in a supine position. A non-contrast CT scan of the chest was performed.

Epicardial EGM reconstruction has been previously described[1]. A 3D mesh of the heart is derived from the CT scan alongside the position of each electrode in the vest. CardioInsight™ software then calculates epicardial EGMs by combining information from the body surface electrodes and the 3D coordinates from the CT derived mesh. Approximately 1,200 epicardial electrograms could be extracted per 3D mesh.

Peak exercise and 10 minutes of recovery were bookmarked at the time of testing. The earliest 10 cardiac cycles considered sufficiently artefact-free for analysis were selected following each of these bookmarks. Heart rates were recorded for each of the sampled segments.

To reduce the effect of random noise, finite impulse response filtering and signal averaging were performed, summarizing each timestrip of 10 cardiac cycles to a single, signal averaged beat. To minimize user bias, electrogram segmentation and curation were fully automated. We developed a convolutional neural network (CNN) based on an existing model to bound QRS complexes and T waves [2]. Our CNN was trained on over 20,000 labelled beats from a separate public database [3]. Once the neural network had estimated the bounds of QRS and T waves for each electrogram, the overall bounds were taken from the $2.5^{th}$ to $97.5^{th}$ percentiles of times to diminish outlier effects. Elimination of electrograms unsuitable for analysis was automated to two pre-specified criteria: T-waves less than 3% the size of the QRS complex or having more than 3 deflections [4].

### 1.1 Analysis of surface ECG markers

To examine for body surface recording signs of conduction pathology, QRS durations were measured for the peak exercise and recovery datasets. The vest output rather than conventional 12-lead was used to avoid timing issues with the exercise machine output. The positional equivalent of 12-lead ECG V2 was used (electrodes 71-76 on the CardioINSIGHT™ vest), with the first beat from the sample as the representative measurement.

### 1.2 Measures of epicardial electrophysiology

For a given epicardial electrogram, local activation time (AT) was defined as the period from QRS start to steepest negative point of the QRS complex, and local activation-recovery intervals (ARI) as

local activation time to steepest positive point of the T wave (Wyatt method[4]). The Wyatt method is favored by ECGi mapping papers to date[5-8].

To search for steep electrical gradients, each electrogram location on the epicardial shell was linked to neighboring locations within a 5mm Euclidean search distance. For each node-neighbor pair, the difference in AT or ARI was divided by the distance between the locations, giving a gradient in milliseconds/millimeter. For each node on the epicardial surface, the mean gradient within a 5mm radius was calculated, and these values were averaged across the epicardial shell to give a whole-heart estimation of steep electrical gradients.

To fully understand the electrophysiology of the three groups, three domains were defined for analysis:

1. The mean of activation or ARI was used to describe overall *conduction* or *repolarization delay*.
2. The central 95% range of times was used to describe *dispersion*.
3. The mean gradient of activation and ARI times in space was used to detect the presence of *steep gradients*.

### 1.3 Logistic regression for the description of the arrhythmogenic substrate in HCM

To understand the contribution of different parameters from our panel to the arrhythmogenic substrate in HCM, we built multiple variable logistic models from significant variables. To qualify for inclusion, a measure would have to significantly differentiate HCM VF and HCM volunteers ($p<0.05$).

Qualifying measures were scaled to the mean and variance of the whole dataset. To improve the ability of the model to predict on unseen data, collinearity was reduced by rejecting one measure of any pair with a Pearson correlation of >0.8[9]. A multiple logistic model was then fitted using Newton's method. Backward stepwise selection was used to reject variables with $p>0.15$[10, 11]. Odds ratios were calculated by exponent of the model coefficients. The predicted probability of an observation falling into the HCM VF group was compared for the true HCM VF group, and the HCM group without previous arrhythmia.

### 1.4 Ability of a multiple logistic model to predict in unseen data

To determine the ability of these logistic models to predict whether a patient was in the HCM or HCM VF group in a wider population, k-folds validation was performed. K-folds validation is used in small datasets because it tests on the entire population ($n$), thereby avoiding the potentially large effect of single outliers in small validation sets[12, 13]. Briefly, a subset of patients is reserved for testing (size $\frac{n}{k}$, a 'fold'), and the remaining patient data is used to train a logistic regression model. The accuracy of this model is then assessed on the reserved testing group. This is repeated by reserving a new testing group and training another model on the remaining data. Once all $k$ folds are tested, the accuracy results are aggregated to estimate sensitivity and specificity.

## 2 Supplementary figure S1

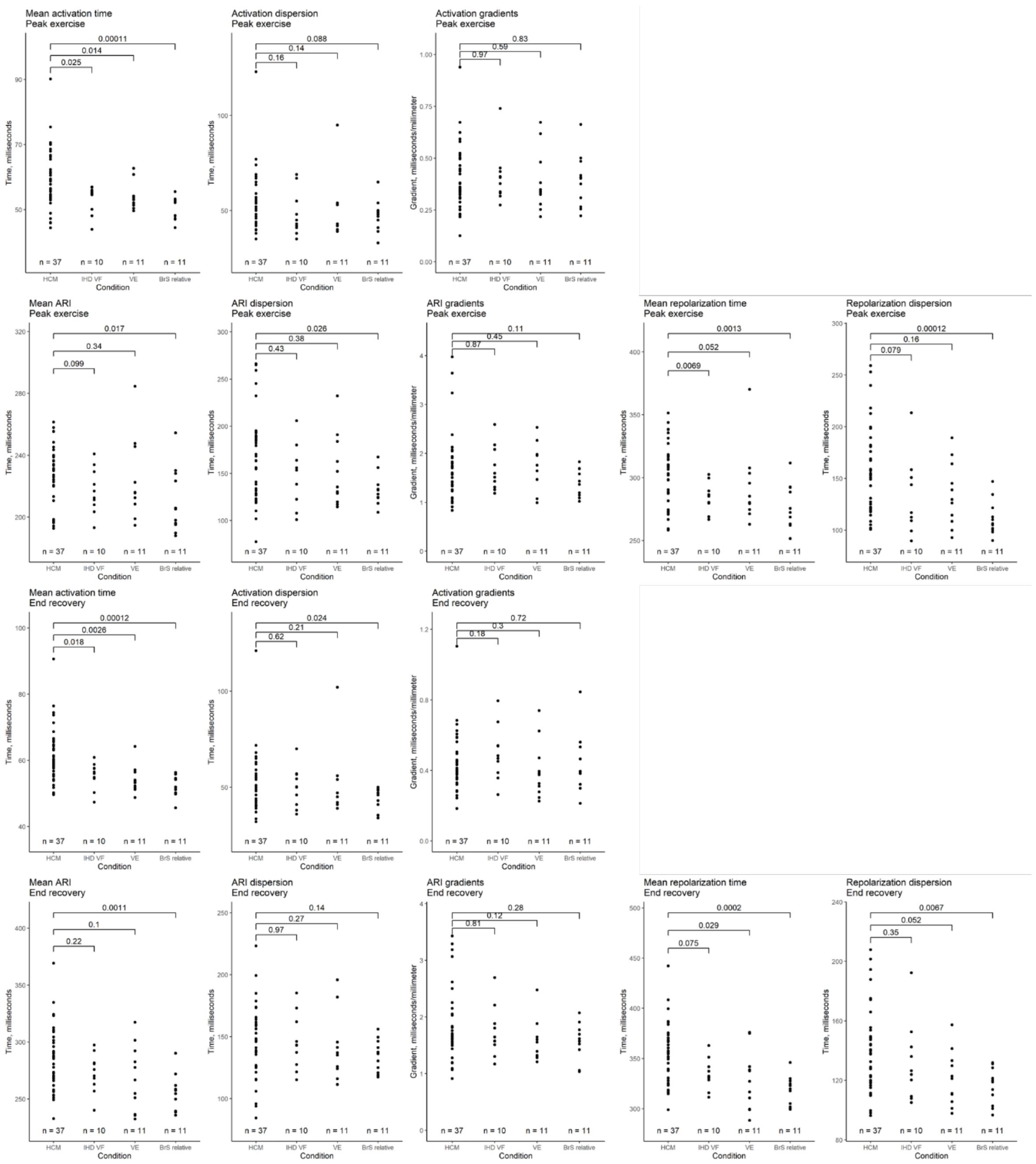


Supplementary figure S1: Comparison of whole heart activation and repolarization metrics immediately after peak exercise and in end recovery between hypertrophic cardiomyopathy (HCM) and a selection of structurally normal heart control groups: (I) fully recovered and revascularized ischaemic VF survivors, IHD VF; (II) patients with benign but symptomatic idiopathic ventricular ectopy, VE; (III) the unaffected relatives of patients with Brugada syndrome, BrS relative. Local activation time (LAT) was defined as the onset of the first epicardial QRS complex to the steepest negative slope of the electrogram-QRS complex. Local repolarization time (LRT) was defined as the onset of the first epicardial QRS complex to the steepest positive slope of the electrogram-T wave. Activation recovery interval (ARI) is the difference between LAT and LRT. Mean time is the average of all LAT/LRT/ARI across the heart. Dispersion is the central 95% range of LAT/LRT/ARI

across the heart. Gradient is the whole-heart mean rate of range in LAT/ARI over a 5mm search distance around each epicardial location.

## 3 Supplementary figure S2

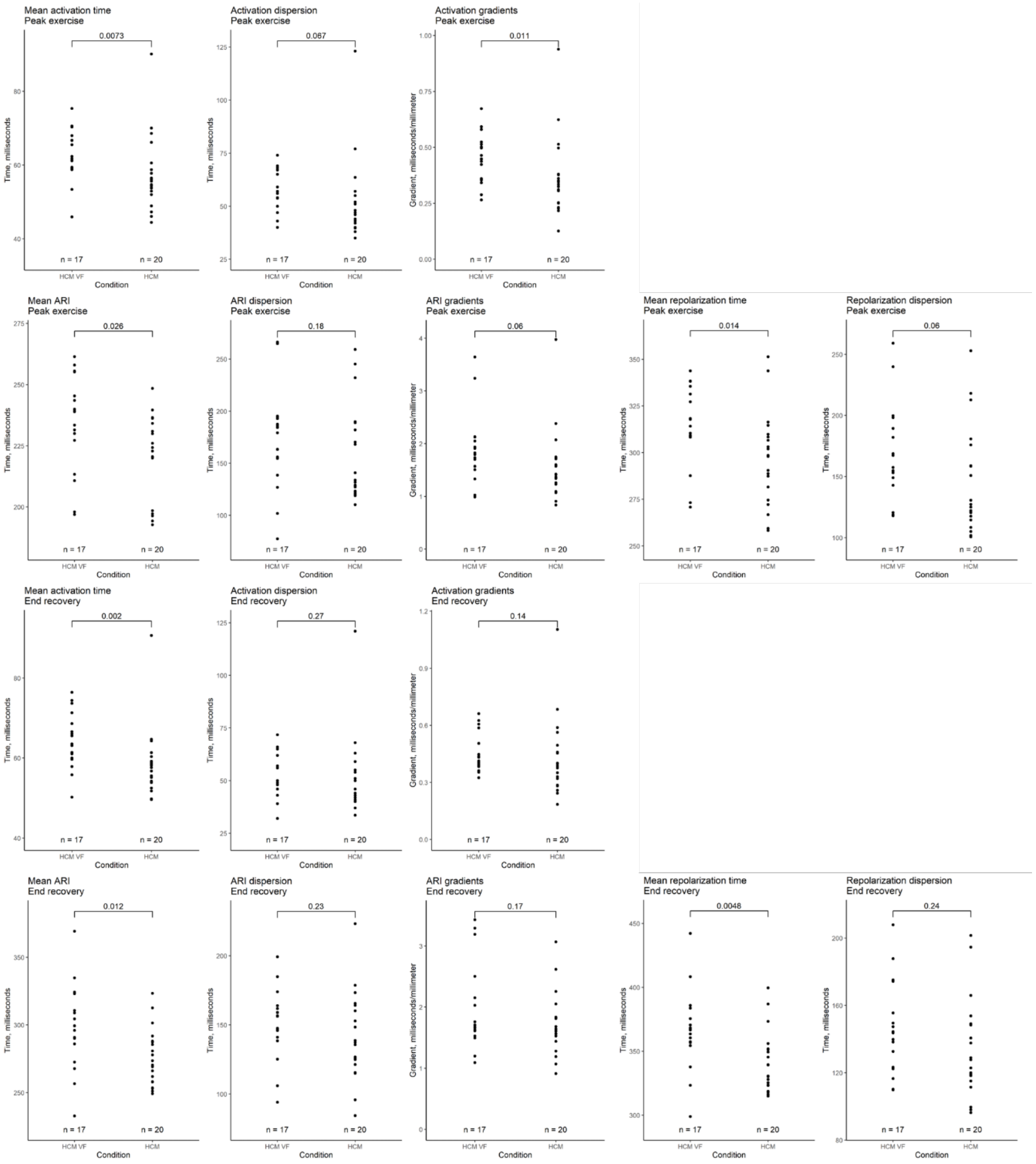


Supplementary figure S2: Comparison of whole heart activation and repolarization metrics immediately after peak exercise and in end recovery between hypertrophic cardiomyopathy (HCM) patients without a personal arrhythmic history and VF or haemodynamically unstable VT survivors (HCM VF). Local activation time (LAT) was defined as the onset of the first epicardial QRS complex to the steepest negative slope of the electrogram-QRS complex. Local repolarization time (LRT) was defined as the onset of the first epicardial QRS complex to the steepest positive slope of the electrogram-T wave. Activation recovery interval (ARI) is the difference between LAT and LRT. Mean time is the average of all LAT/LRT/ARI across the heart. Dispersion is the central 95% range

of LAT/LRT/ARI across the heart. Gradient is the whole-heart mean rate of range in LAT/ARI over a 5mm search distance around each epicardial location.

## 4 Supplementary table S1

Table S1: Correlation matrix to detect intervariable dependence. High Pearson correlation between two variables suggests a 1:1 relationship and predisposes models to collinearity. In our study we chose to eliminate one of any pair of variables more with a Pearson correlation >0.8 (high inter-dependence). In this case, mean activation time in exercise was eliminated (high correlation with mean activation time in recovery). Activation recovery interval, ARI.

| | **Recovery mean activation time** | **Exercise mean activation time** | **Recovery mean ARI** | **Exercise mean ARI** | **Exercise activation gradients** |
|---|---|---|---|---|---|
| **Recovery mean activation time** | 1.00 | 0.93 | 0.23 | 0.32 | 0.72 |
| **Exercise mean activation time** | 0.93 | 1.00 | 0.21 | 0.34 | 0.72 |
| **Recovery mean ARI** | 0.23 | 0.21 | 1.00 | 0.57 | 0.21 |
| **Exercise mean ARI** | 0.32 | 0.34 | 0.57 | 1.00 | 0.31 |
| **Exercise activation gradients** | 0.72 | 0.72 | 0.21 | 0.31 | 1.00 |

## 5 Supplementary table S2

| 4-variable model regression results | | | | | | |
|---|---|---|---|---|---|---|
| No. Observations: | 37 | | | | | |
| Df Residuals: | 32 | | | | | |
| Df Model: | 4 | | | | | |
| Pseudo R-squared: | 0.1930 | | | | | |
| Log-Likelihood: | -20.600 | | | | | |
| LL-Null: | -25.525 | | | | | |
| LLR p-value: | 0.04302 | | | | | |
| | **coefficient** | **SE** | **z-value** | **P>\|z\|** | **[0.025** | **0.975]** |
| constant | -14.7861 | 6.239 | -2.370 | 0.018 | -27.015 | -2.557 |
| Recovery mean activation time | 0.0784 | 0.071 | 1.102 | 0.270 | -0.061 | 0.218 |
| Recovery mean ARI | 0.0283 | 0.019 | 1.476 | 0.140 | -0.009 | 0.066 |
| Exercise mean ARI | 0.0062 | 0.027 | 0.232 | 0.816 | -0.046 | 0.059 |
| Exercise mean activation gradients | 0.8177 | 3.674 | 0.223 | 0.824 | -6.383 | 8.019 |

Supplementary table S2: Regression parameters for 4-variable model differentiating hypertrophic cardiomyopathy patients with and without a personal history of life-threatening arrhythmia. Standard error of the coefficient, SE; coefficient divided by standard error, z-value; significance of the coefficient, P>|z|; lower and upper 95% confidence bound, [0.025 0.975].

## 6 Supplementary table S3

| 2-variable model regression results | | | | | | |
|---|---|---|---|---|---|---|
| No. Observations: | 37 | | | | | |
| Df Residuals: | 34 | | | | | |
| Df Model: | 2 | | | | | |
| Pseudo R-squared: | 0.1904 | | | | | |
| Log-Likelihood: | -20.665 | | | | | |
| LL-Null: | -25.525 | | | | | |
| LLR p-value: | 0.007754 | | | | | |
| | **coefficient** | **std err** | **z-value** | **P>\|z\|** | **[0.025** | **0.975]** |
| constant | -14.6505 | 5.667 | -2.585 | 0.010 | -25.758 | -3.543 |
| Recovery mean activation time | 0.0918 | 0.057 | 1.614 | 0.107 | -0.020 | 0.203 |
| Recovery mean ARI | 0.0311 | 0.016 | 1.963 | 0.050 | 4.95e-05 | 0.062 |

Supplementary table S3: Regression parameters for 2-variable model differentiating hypertrophic cardiomyopathy patients with and without a personal history of life-threatening arrhythmia. Standard error of the coefficient, SE; coefficient divided by standard error, z-value; significance of the coefficient, P>|z|; lower and upper 95% confidence bound, [0.025 0.975].

## 7 Results of a surface measures only logistic regression model

To ascertain the incremental value of ECG imaging over the surface ECG in identifying VF survivors from the HCM cohort, the logistic regression analysis was repeated using only QRS duration and QTc in both exercise and recovery. The both the balanced accuracy and area under the receiver operating characteristic curve of the 2-variable ECGi model was superior to the surface measures only model.

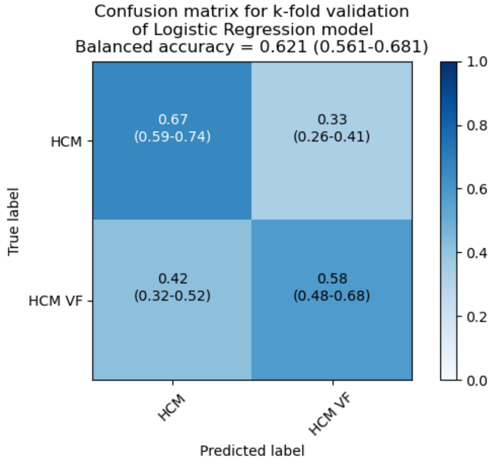


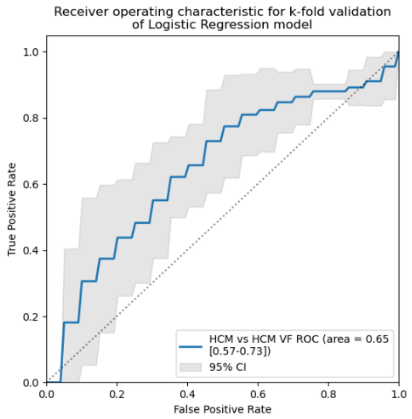


| Surface data model regression results | |
|---|---|
| **No. Observations:** | 37 |
| **Df Residuals:** | 32 |
| **Df Model:** | 4 |
| **Pseudo R-squ.:** | 0.1663 |
| **Log-Likelihood:** | -21.280 |
| **LL-Null:** | -25.525 |
| **LLR p-value:** | 0.07521 |

| | **coefficient** | **std err** | **z** | **P>\|z\|** | **[0.025** | **0.975]** |
|---|---|---|---|---|---|---|
| constant | -12.2153 | 5.563 | -2.196 | 0.028 | -23.118 | -1.313 |
| Recovery QRSD | -0.0354 | 0.032 | -1.117 | 0.264 | -0.097 | 0.027 |
| Exercise QRSD | 0.0170 | 0.025 | 0.673 | 0.501 | -0.032 | 0.066 |
| Recovery QTc | 0.0399 | 0.016 | 2.432 | 0.015 | 0.008 | 0.072 |
| Exercise QTc | -0.0066 | 0.014 | -0.477 | 0.633 | -0.034 | 0.020 |

# 8 Detailed characteristics of the condition groups

## 8.1 Table S4: Ischaemic VF

| | |
|---|---|
| Count | 10 |
| Age (years) | 58.3 ±8.0 |
| Sex M:F | 9:1 |

| | |
|---|---|
| VF during presentation | 10/10 |
| Anginal symptoms in year prior to testing | 0/10 |
| ECG at testing | 3 normal sinus rhythm<br>3 residual ST elevation (<1mm)<br>1 anterior early repolarization<br>3 residual T wave inversion |
| MRI | Performed in 1, late Gad enhancement of papillary muscle but no regional motion abnormality |
| Normal LV function, no regional wall motion abnormalities on echocardiogram | 10/10 |
| Infarct location/revascularization | 2 LAD<br>2 Circumflex/OM<br>2 RCA/PDA<br>4 triple vessel disease |
| Mean peak troponin I (ng/L) | 15387 ±20919 |

## 8.2 Table S5: Brugada relatives

| | |
|---|---|
| Count | 11 |
| Age (years) | 45.5 ±11.0 |
| Sex M:F | 8:3 |
| ECG at testing | 8 normal sinus rhythm<br>1 RSR' in V1-2 |

| | |
|---|---|
| | 1 early repolarization in V1-2<br>1 W pattern in V1 |
| Echocardiogram | 10 normal<br>1 bicuspid aortic valve, otherwise normal |
| MRI | Performed in 1, normal |
| Coronary assessment | 10 normal ETT<br>1 normal ETT and DSE |
| Negative Ajmaline challenge | 11/11 |
| Family history | 4 (aborted) sudden death<br>4 spontaneous Type 1 ECG<br>3 concealed Type 1 ECG |

### 8.3 Table S6: Ventricular ectopy ablation

| | |
|---|---|
| Count | 11 |
| Age (years) | 44.5 ±14.3 |
| Sex M:F | 6:5 |
| ECG (excluding ectopy) | 10 normal sinus rhythm<br>1 anterior T wave abnormality |
| Echocardiogram | 10 normal<br>1 mitral valve prolapse, otherwise normal |
| MRI | 3 normal<br>2 mild LV dysfunction in the context of ectopy without evidence of fibrosis |

| | |
|---|---|
| | 6 not performed |
| Ectopy location | 8 RVOT<br>1 LVOT<br>1 Basal septal LV<br>1 inferior LV |
| Ectopy burden | 17.2 ±10.2% |

### 8.4 Table S7: Hypertrophic cardiomyopathy with VF/VT

| | |
|---|---|
| Count | 17 |
| Age (years) | 45.5 ±14.8 |
| Sex M:F | 13:4 |
| Time from HCM diagnosis (years) | 4.3 ±3.4 |
| Time from arrhythmic event (years) | 3.5 ±3.5 |
| ECG | 7 lateral T wave inversion<br>3 lateral or inferolateral ST depression<br>3 inferolateral or inferior T wave inversion<br>2 left ventricular hypertrophy voltage criteria<br>1 left bundle branch block<br>1 poor R wave progression |
| MRI Late Gadolinium enhancement (LGE) burden | 1 with >15% or 'extensive' LGE<br>1 with <15% or 'non-extensive' or 'mild' LGE |

| | |
|---|---|
| | 3 with LGE but no quantification<br><br>1 without LGE<br><br>11 without MRI/LGE sequence performed |
| Genetics<br><br>(Pathogenic unless stated otherwise) | 4 MYBPC3<br><br>3 TNNI3<br><br>1 MYH7<br><br>1 TPM1<br><br>8 did not undergo genetic testing |

### 8.5 Table S8: Hypertrophic cardiomyopathy without life threatening arrhythmia

| | |
|---|---|
| Count | 20 |
| Age (years) | 52.0 ±13.9 |
| Sex M:F | 15:5 |
| Time from HCM diagnosis (years) | 2.8 ±2.3 |
| ECG | 8 lateral T wave inversion<br><br>4 inferior and lateral T wave inversion<br><br>4 normal sinus rhythm<br><br>2 left bundle branch block<br><br>1 poor R-wave progression<br><br>1 left ventricular hypertrophy voltage criteria without T wave inversion |
| MRI Late Gadolinium enhancement (LGE) burden | 8 with >15% or 'extensive' LGE<br><br>6 with <15% or 'non-extensive' or 'mild' LGE |

| | |
|---|---|
| | 3 with LGE but no quantification<br>1 without LGE<br>2 without MRI/LGE sequence performed |
| Genetics<br>(Pathogenic unless stated otherwise) | 4 MYBPC3 (1/4 VUS)<br>1 TNNI3<br>1 TNNT2<br>7 without detection of known causative gene<br>7 did not undergo genetic testing |

## 9 Inter-operator reproducibility

The software used for analysis was written by two of the study authors (J. C. and M. S-S.). To determine whether an early stage user of the software could reproduce the measurements, an inter-operator reproducibility study was performed.

R. G. (see acknowledgements), a first-year cardiology registrar (5 years post qualification as a doctor) was recruited as the trainee. J. C. delivered approximately 2 hours of in-person teaching and the trainee was allowed to experiment making measurements through the software unsupervised.

An early issue raised by the trainee was failure of the neural network to segment the T-wave correctly. It was found that the trainee had set the QRST window of interest to include some of the preceding T wave, leading to the neural network highlighting the area from the end of the last QRST to the end of the target QRST. Care to avoid the previous QRST complex allowed normal function of the software. This provided a useful cue to the trainee for when a QRST selection deviated from the ideal.

Following this learning point, the trainee (Operator 1) performed measurements on 10 HCM patients, blinded to the scores of the present study's author (Operator 2). Spearman's R was >0.9 for both AT and ARI measurements, suggesting that even for a new user of the software, reproducible measurements are possible.

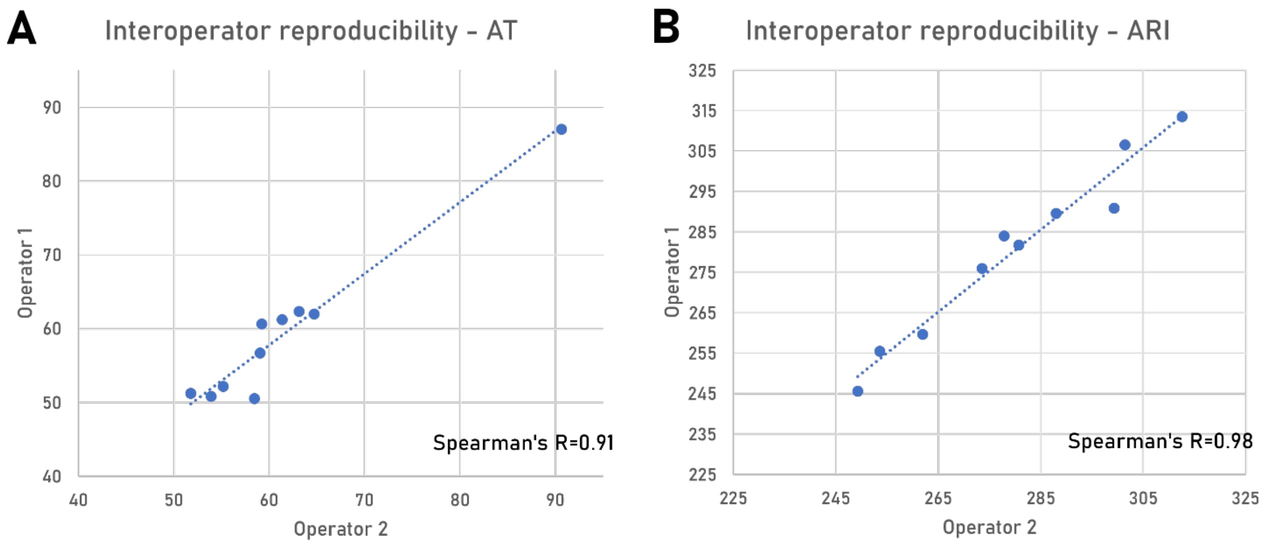


Graphs have been adapted with permission from Dr Rahul Ghelani, clinical research fellow at the National Heart and Lung Institute, Imperial College London, UK.

## 10 References for the supplement


1. Rudy Y, Lindsay BD. Electrocardiographic imaging of heart rhythm disorders: from bench to bedside. Cardiac electrophysiology clinics 2015;7:17-35.
2. Jia D, Zhao W, Li Z, Hu J, Yan C, Wang H, You T. An Electrocardiogram Delineator via Deep Segmentation Network. Paper presented at: 2019 41st Annual International Conference of the IEEE Engineering in Medicine and Biology Society (EMBC)2019.
3. Kalyakulina AI, Yusipov II, Moskalenko VA, Nikolskiy AV, Kozlov AA, Kosonogov KA, Zolotykh NY, Ivanchenko MV. LU electrocardiography database: a new open-access validation tool for delineation algorithms. arXiv preprint arXiv:180903393 2018.
4. Wyatt RF, Burgess MJ, Evans AK, Lux RL, Abildskov JA, Tsutsumi T. Estimation of ventricular transmembrane action potential durations and repolarization times from unipolar electrograms. The American journal of cardiology 1981;47:488.
5. Leong KMW, Ng FS, Roney C, et al. Repolarization abnormalities unmasked with exercise in sudden cardiac death survivors with structurally normal hearts. Journal of cardiovascular electrophysiology 2018;29:115-126.
6. Zhang J, Hocini M, Strom M, Cuculich PS, Cooper DH, Sacher F, Haïssaguerre M, Rudy Y. The Electrophysiological Substrate of Early Repolarization Syndrome: Noninvasive Mapping in Patients. JACC: Clinical Electrophysiology 2017;3:894-904.
7. Zhang J, Sacher F, Hoffmayer K, et al. Cardiac electrophysiological substrate underlying the ECG phenotype and electrogram abnormalities in Brugada syndrome patients. Circulation 2015;131:1950-1959.
8. Andrews CM, Srinivasan NT, Rosmini S, Bulluck H, Orini M, Jenkins S, Pantazis A, McKenna WJ, Moon JC, Lambiase PD, Rudy Y. Electrical and Structural Substrate of Arrhythmogenic Right Ventricular Cardiomyopathy Determined Using Noninvasive Electrocardiographic Imaging and Late Gadolinium Magnetic Resonance Imaging. Circulation Arrhythmia and electrophysiology 2017;10.
9. Mason CH, Perreault WD. Collinearity, Power, and Interpretation of Multiple Regression Analysis. Journal of Marketing Research 1991;28:268-280.

**10.** Hosmer Jr DW, Lemeshow S, Sturdivant RX. Applied logistic regression. Vol 398: John Wiley & Sons; 2013.
**11.** Chowdhury MZI, Turin TC. Variable selection strategies and its importance in clinical prediction modelling. Family Medicine and Community Health 2020;8:e000262.
**12.** Kim J-H. Estimating classification error rate: Repeated cross-validation, repeated hold-out and bootstrap. Computational statistics & data analysis 2009;53:3735-3745.
**13.** Kohavi R. A study of cross-validation and bootstrap for accuracy estimation and model selection. Paper presented at: Ijcai1995.
